# Increased Serum Lipid Levels in Patients with Subjective Tinnitus 

**DOI:** 10.22038/ijorl.2020.43663.2442

**Published:** 2021-01

**Authors:** Deniz Avcı

**Affiliations:** *Department of Otorhinolaryngology, Nevsehir State Hospital, Nevsehir, Turkey.*

**Keywords:** HDL, LDL, Subjective tinnitus, Total cholesterol, Triglyceride

## Abstract

**Introduction:**

The aim was to investigate the link between tinnitus and serum levels of total cholesterol (TC), triglyceride (TRG), low-density (LDL) and high-density lipoprotein (HDL) in the central anatolian Turkish population.

**Materials and Methods:**

The retrospective and case-control study included a total of 91 patients with subjective tinnitus and a control group of age- and sex-matched 65 healthy volunteers. A detailed otolaryngologic examination followed by pure tone audiometry, serum lipid values, and magnetic resonance imaging of the temporal bone was performed. The clinical characteristics of tinnitus were registered for all patients. The serum levels of TC, TRG, LDL and HDL were compared between the two groups.

**Results:**

Mean TC level was 200.57±41.06 mg/dL in the patient group and 179.0±39.03 mg/dL in the control group (P=0.001). Mean TRG level was 177.76±86.94 mg/dL in the patient group and 124.43±61.44 mg/dL in the control group (P=0.000). Mean LDL level was 115.88±32.56 mg/dL in the patient group and 101.31±34.42 mg/dL in the control group (P=0.008). Mean HDL level was 50.25±13.60 mg/dL in the patient group and 53.46±12.66 mg/dL in the control group (P=0.137). Among all the serum lipids, TC, TRG and LDL established a significant difference between the two groups.

**Conclusion:**

The results indicated that TC, TRG and LDL levels were significantly higher in tinnitus group and this increase implicates the potential role of hyperlipidemia associated with altered lipid metabolism in the etiology of tinnitus. We suggest that serum lipid levels could be useful and conducive in the diagnosis and prognosis of tinnitus.

## Introduction

Tinnitus is a situation of the ear characterized by the insight of sounds that cannot be based on an external source. Tinnitus is not a clinical entity alone and has an unknown pathophysiology although it has been based on anatomical and/or functional changes in the hearing system. The prevalence of tinnitus in both sexes has been presented to be 10.1-14.6% and the prevalence has been expected to increase with age. In young adults, tinnitus has been connected with depression, sleep deprivation, anxiety, and diminished quality of life (1). Tinnitus can be subjective, objective, pulsatile or nonpulsatile based on its clinical characteristics. Idiopathic type subjective tinnitus is the most pervasive type of tinnitus (2). Inner ear is an end organ that supplies blood for stria vascularis and hair cells and is highly susceptible to vascular events. Hyperlipidemia leads to accumulation of lipids in end arteries, causing narrowing, thereby resulting in reduced blood supply, ultimately causing chronic hypoxia that impairs cochlear metabolism. This mechanism may cause accumulation of free radicals due to its adverse effect on the capability of antioxidant enzymes. As a result, peroxidative damage may occur in the hair cells of the cochlea and stria vascularis due to compromised oxygen supply (3). The changes of blood flow in cochlea and the limited impairment of perfusion results in dysfunction of the organ of corti, thereby leading to tinnitus. Hyperlipidemia may also cause tinnitus by compromising cochlear blood flow, and tinnitus may be the initial symptom of atherosclerosis (4). To our knowledge, there is little documentation of the prevalence and mechanism of dyslipidemia in tinnitus in the internet-based literature. In the present study, in the central anatolian Turkish population, we purposed to assess the connection between subjective tinnitus and the serum levels of total cholesterol (TC), triglyceride (TRG), low-density lipoprotein (LDL), and high-density lipoprotein (HDL) based on the hypothesis that hyperlipidemia can be a cause of subjective tinnitus and also to discuss our findings in light of studies in the literature, which are highly rare.

## Materials and Methods

The retrospective case-control study contained 91 patients and 65 healthy individuals that presented to Otorhinolaryngology Department with a one-month background of subjective tinnitus complaint between October 2018 and May 2019. The research article was approved by University’s Ethics Board. Helsinki Declaration rules were applied in the study. Informed written consent form is not necessary due to retrospective study. 

The tinnitus group included 91 patients, including 57 (62.6%) women and 34 (37.4%) men with a mean age of 48.03±15.12 years. The control group included 65 healthy subjects, including 41 (63.1%) women and 24 (36.9%) men with a mean age of 47.55±17.49 years.

A detailed otolaryngological examination followed by pure tone audiometry (PTA), tympanometry, serum lipid values, and magnetic resonance imaging (MRI) of the temporal bone was performed. The clinical characteristics and time of tinnitus were registered for patients suffer from unilateral or bilateral tinnitus. The level of subjective tinnitus was evaluated by using the Turkish validity of Tinnitus Handicap Inventory (THI) (5). The 25 questions of THI were asked to patients. Patients were distributed to 5 different groups with respect to THI scores (Stage 1= 0-16 score= Slight, Stage 2= 18-36 score= Mild, Stage 3= 38-56 score=Moderate, Stage 4= 58-76 score= Severe, Stage 5= 78-100 score= Catastrophic) (5).

Patients with all kinds of outer, middle, or inner ear disease, all kinds of hearing loss, uncontrolled systemic diseases, malignancies, acute and chronic inflammatory diseases, presence of acoustic trauma, and patients with aged below 18 or over 70 years, were excluded from the study. Patients who had non-pulsatile, subjective tinnitus graded as slight, mild, moderate, severe, or catastrophic based on the THI scores. Patients and healthy volunteers with normal air and bone thresholds, laboratory and otolaryngologic examination findings were included in the study. We eliminate differential diagnosis by MRI only in all patients. All the PTA and tympanometric evaluations were applied by the same audiometry crew using an Interacoustics AC-40 clinical audiometer (Assens, Denmark) and 226 Hertz (Hz) Maico tympanometer (Berlin, Germany). PTA was evaluated air thresholds at 250, 500, 1000, 2000, 4000, and 8000 Hz. and bone sills at 500, 1000, 2000, and 4000 Hz. Pure tone average was accepted as the threshold value averaged across 0.5, 1, 2, and 4 kilohertz (kHz). Patients with Type A tympanometry (ranging from +50 daPa to -50 daPa) were included in this study.

**Statistical analysis**

Data were investigated by SPSS 22.0 program (IBM, Armonk, NY, USA). Descriptives were characterized as mean and standard deviation (SD) for numerical variables, and expressed as frequencies and percentages for categorical variables. Numerical variables that follow parametric assumptions were evaluated using Independent samples t-test and categorical variables were evaluated using Chi-square test. Parametric assumptions were controlled by Shapiro-Wilk test. The normality of the tests was done using Skewness and Kurtosis values. Since all values were between -1.5 and +1.5, it was observed that they were normally distributed. The serum levels of TC, TRG, LDL, and HDL were compared between tinnitus and control groups. A p value of <0.05 was accepted significant.

## Results

No significant difference was detected between the tinnitus and control groups with regard to age (P=0.855) and sex (P=0.956) (Table.1). Mean time of presence of tinnitus was 7.17±10.52 months in the patient group. Of the 91 patients, 32 (35.2%) patients had right ear tinnitus, 35 (38.5%) patients had left ear tinnitus, and 24 (26.3%) patients had bilateral tinnitus. In the patient group, mean PTA threshold value was 17.01±6.57 dB HL (decibel hearing level) in the right ear and 16.05±6.05 dB HL in the left ear and no significant difference was found between the ears in point of PTA threshold value (P>0.05). Mean THI point was 37.29±15.22 in the patient group, which corresponded to stage 3 tinnitus(Table 2). 

**Table 1:** Comparison of demographic and clinical features of tinnitus and control groups (Independent Sample T test and Chi-Square test were used)

**Variables**	**Tinnitus Group** **(n=91)** **Mean** **±SD**	**Control Group** **(n=65)** **Mean** **±SD**	**p value**	**Tests of Normality** **S** ** / ** **K**
Age	48.03±15.12	47.55±17.49	0.855	
Gender				
Female	57 (62.6%)	41 (63.1%)		
Male	34 (37.4%)	24 (36.9%)	0.956	
Total Cholesterol (TC) (mg/dL)	200.57±41.06	179.0±39.03	0.001*	0.57±0.19 / 0.13±0.38
Triglyceride (TRG) (mg/dL)	177.76±86.94	124.43±61.44	0.000*	1.02±0.19 / 0.84±0.38
High Density Lipoprotein (HDL) (mg/dL)	50.25±13.60	53.46±12.66	0.137	0.72±0.19 / 1.11±0.38
Low Density Lipoprotein (LDL) (mg/dL)	115.88±32.56	101.31±34.42	0.008*	0.61±0.19 / 0.00±0.38

**SD**: Standard deviation, **S:** Skewness, **K:** Kurtosis,

* Statistically significant

**Table 2:** Clinical data of the patients in tinnitus group (n=91)

Tinnitus side	Right		32 (35.2%)
	Left		35 (38.4%)
	Bilateral		24 (26.4%)
			**Mean±SD**
THI Average			37.29±15.22
PTA (dB HL)	Right Left		17.01±6.57 16.05±6.05
		Tinnitus Time (Month) 7.17±10.52	

SD: Standard deviation, dB HL: Decibels hearing level, THI: Tinnitus Handicap Inventory, PTA: Pure tone average

**Fig 1:**
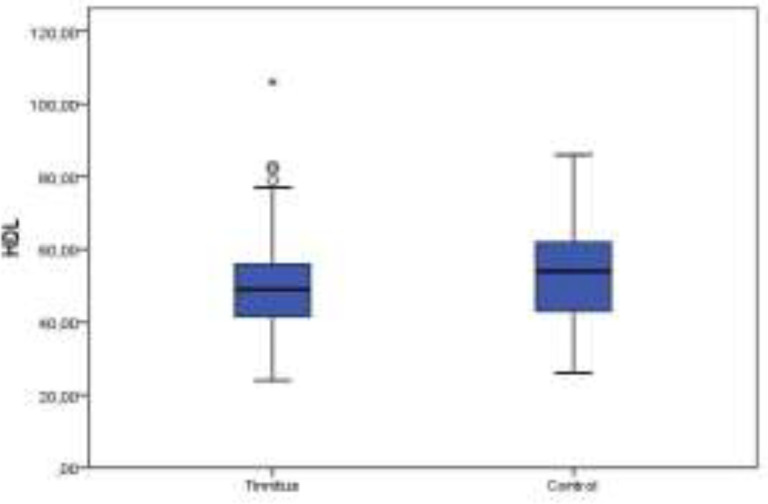
Distribution of high-density lipoprotein (HDL) levels between tinnitus and control groups on boxplot graph

**Fig 2:**
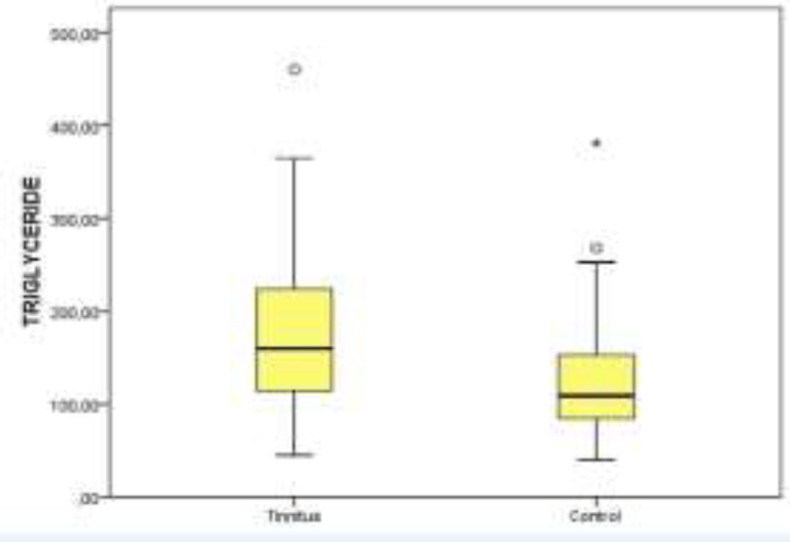
Distribution of triglyceride levels between tinnitus and control groups on boxplot graph

**Fig 3:**
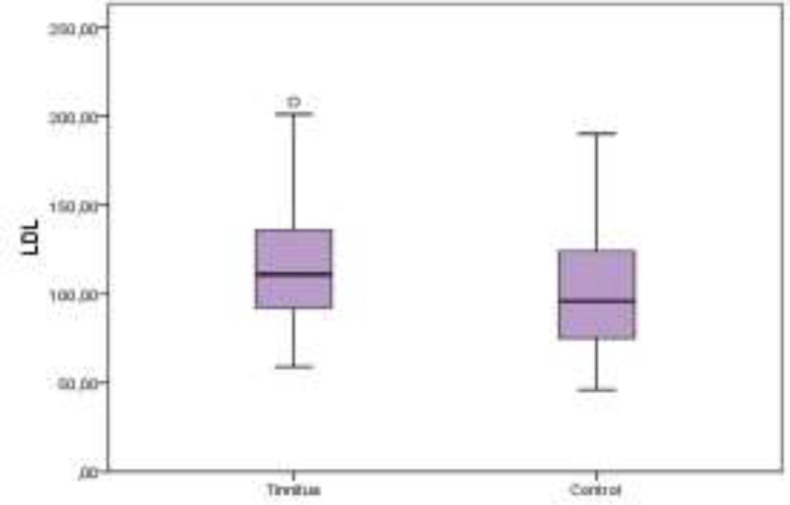
Distribution of low-density lipoprotein (LDL) levels between tinnitus and control groups on boxplot graph

**Fig 4:**
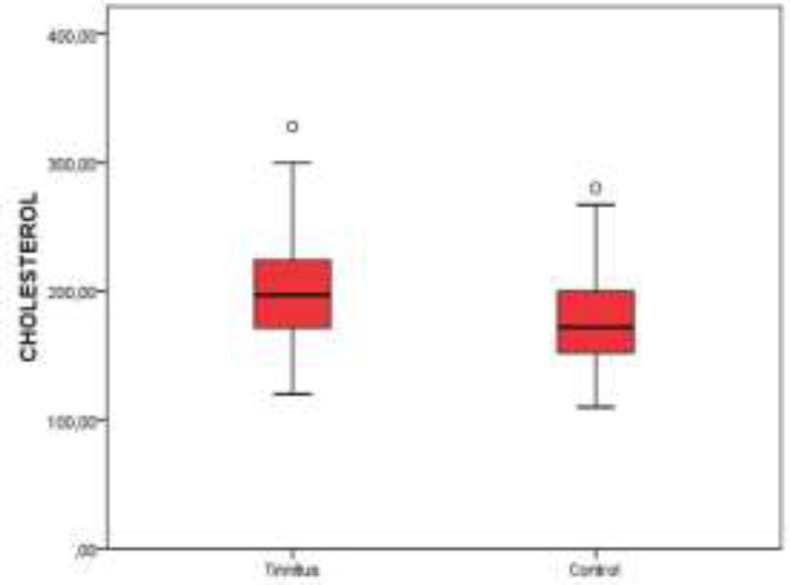
Distribution of total cholesterol levels between tinnitus and control groups on boxplot graph

Table 1 offers the demographic features and serum lipid levels of both groups. No significant difference was detected between the tinnitus and control groups in point of HDL levels (P>0.05) (Fig.1). The serum levels of TC, TRG, and LDL were significantly higher in the tinnitus group (P<0.05) (Fig. 2-4). In the tinnitus group, TC and TRG levels were >200 mg/dL in 44 (48.3%) and 30 (33.0%) patients, respectively. In the same group, LDL level was >130 mg/dL in 28 (31.0%) patients and HDL level was <45 mg/dL in 38 (41.7%) patients. 

## Discussion

In spite of its elevated prevalence, tinnitus has an unclear etiology. Nevertheless, tinnitus has been noticed after surgery, acoustic trauma, damage to the auditory system, inflammation, and ischemia (1). Additionally, tinnitus has been related to the changes at the peripheral and central levels of the auditory system. The level of tinnitus has been noticed to be aggravated by stress. Serotonin plays a crucial role in the sensorial system and auditory streaming and serotonin discharge has been indicated to be increased in some brain areas during stress. Therefore, serotonin dysfunction may also have a role in the improvement of tinnitus (6).

The universal goal of studies investigating tinnitus is to elucidate the pathophysiological mechanisms of tinnitus. Recently, dyslipidemia has been reported as an etiology of tinnitus (7). Some other studies also showed that a hyperlipidemic diet may damage stria vascularis, cochlear apical turn and inner ear outer hair cells and that hypercholesterolemia may lead to tinnitus (8). In hyperlipidemia, vascular factors alone are not responsible for hearing disorders. Norena et al. detected water-soluble cholesterol in the outer hair cells of Guinea pigs and reported it as a foreign body which could be an alternative mechanism of lipid deposition in membranous inner ear. The authors also noted that this foreign body reaction and this acquisition of cholesterol can be accompanied by transduction abnormalities and increased cell stiffness, both of which disrupt cell electromotility (9). Cai et al. showed that simvastatin treatment preserved the hearing functions of rats with apolipoprotein E-deficiency that were given a high-fat diet. They attributed this effect to the treatment of hyperlipidemia (10). Satar et al. found that hypercholesterolemia affects negatively the stria vascularis and hair cells, thereby causing hearing loss and transduction abnormalities (8). Hameed et al. evaluated 51 patients and showed a relationship between tinnitus and hyperlipidemia based on the finding that revealed that atorvastatin treatment decreased serum cholesterol levels and also improved tinnitus scores (11). Similarly, Pulec et al. reported that the tinnitus scores improved by 83% among the patients treated with hyperlipidemic diet and also noted that the elevated serum lipid levels may lead to inner ear dysfunction. The authors attributed this condition to the relationship between hyperlipidemia and tinnitus (12). On the contrary, Canis et al. detected no improvement in tinnitus scores in patients with hyperlipidemia that were treated with simvastatin for a period of 4 months (13). In line with these data, we also found that increased serum lipid levels may have a significant role in the etiology of tinnitus. On the other hand, some previous histochemical studies that were conducted with hypercholesterolemic animals reported vacuolar degeneration in stria vascularis and patches of an amorphous substance in outer hair and strial cells (14). Cholesterol may disrupt cochlear microcirculation structure by reducing the endothelial expression of nitric oxide, which is a potent vasodilator, and by increasing blood viscosity. The cause and effect connection between hyperlipoproteinemia and inner ear disease is indisputable. Hyperlipoproteinemia may cause spasm of the vestibulocochlear and spiralis modiolic artery (15).

Literature indicates that increased blood fats, obesity, and malnutrition account for 5.1% of the cases of tinnitus, sensorineural hearing loss, and vertigo (12). Based on this data, it is wise to consider that hyperlipoproteinemia is probably the primary cause of chronic obstruction in the capillaries of stria vascularis caused by the biochemical changes and ischemia in the endolymphatic sac. 

Despite being a controversial issue, Oiticica et al. suggested that hearing functions can be affected by the lipid metabolism. The authors also reported hypercholesterolemia in 46-57% of patients with cohcleovestibular dysfunction and tinnitus (16).

Shirazi et al. determinated no significant difference in the prevalence of dyslipidemia between patients with tinnitus and normal individuals (17). In contrast, Yüksel et al. and Yan et al. reported similarly to our study, that the incidence of dyslipidemia and serum levels of TRG, LDL, and TC were significantly higher in tinnitus patients compared to normal individuals (4,15). Also, Cai et al. suggested that the tinnitus intensity returns to normal when serum lipid level is lowered (10). Although the findings of our study contradicted with those of Shirazi et al., they supported the findings presented by Yüksel et al., Yan et al. and Pulec et al. This difference could be attributed to the fact that none of the previous studies performed an evaluation of postprandial serum lipid and diet profiles that could have a role in inner ear functions and tinnitus.

## Conclusion

The results indicated that TC, TRG, and LDL levels were significantly higher in patients with subjective tinnitus in the central anatolian Turkish population and this increase implicates the potential role of hyperlipidemia associated with altered lipid metabolism in the etiology of tinnitus. These parameters can be used as a novel marker of tinnitus. In light of these findings, we suggest that serum lipid levels could be useful in the routine clinical diagnosis and prognosis of subjective tinnitus and the patients detected with dyslipidemia should be treated appropriately.
